# Hyperekplexia, microcephaly and simplified gyral pattern caused by novel *ASNS* mutations, case report

**DOI:** 10.1186/s12883-016-0633-0

**Published:** 2016-07-15

**Authors:** Mohammed Zain Seidahmed, Mustafa A. Salih, Omer B. Abdulbasit, Abdulmohsen Samadi, Khalid Al Hussien, Abeer M. Miqdad, Maha S. Biary, Anas M. Alazami, Ibrahim A. Alorainy, Mohammad M. Kabiraj, Ranad Shaheen, Fowzan S. Alkuraya

**Affiliations:** Neonatology Unit, Department of Pediatrics, Security Forces Hospital, Riyadh, 11481 Saudi Arabia; Division of Pediatric Neurology, Department of Pediatrics, College of Medicine, King Saud University, Riyadh, Saudi Arabia; Pediatric Neurology, Department of Pediatrics, Security Forces Hospital, Riyadh, Saudi Arabia; Developmental Genetics Unit, Department of Genetics, King Faisal Specialist Hospital and Research Center, Riyadh, Saudi Arabia; Department of Radiology and Diagnostic Imaging, King Khalid University Hospital and College of Medicine, King Saud University, Riyadh, Saudi Arabia; Division of Clinical Neurophyisoloy, Department of Neuroscience, Prince Sultan Medical City, Riyadh, Saudi Arabia; Department of Anatomy and Cell Biology, College of Medicine, Al Faisal University, Riyadh, Saudi Arabia

**Keywords:** Hyperekplexia, Brain malformation, Asparagine synthetase deficiency, *ASNS* gene, Whole exome sequencing, Arthrogryposis, Case report

## Abstract

**Background:**

Asparagine synthetase deficiency (OMIM# 615574) is a very rare newly described neurometabolic disorder characterized by congenital microcephaly and severe global developmental delay, associated with intractable seizures or hyperekplexia. Brain MRI typically shows cerebral atrophy with simplified gyral pattern and delayed myelination. Only 12 cases have been described to date. The disease is caused by homozygous or compound heterozygous mutations in the *ASNS* gene on chromosome 7q21.

**Case presentation:**

Family 1 is a multiplex consanguineous family with five affected members, while Family 2 is simplex. One affected from each family was available for detailed phenotyping. Both patients (Patients 1 and 2) presented at birth with microcephaly and severe hyperekplexia, and were found to have gross brain malformation characterized by simplified gyral pattern, and hypoplastic cerebellum and pons. EEG showed no epileptiform discharge in Patient 2 but multifocal discharges in patient 1. Patient 2 is currently four years old with severe neurodevelopmental delay, quadriplegia and cortical blindness. Whole exome sequencing (WES) revealed a novel homozygous mutation in *ASNS* (NM_001178076.1) in each patient (c.970C > T:p.(Arg324*) and c.944A > G:p.(Tyr315Cys)).

**Conclusion:**

Our results expand the mutational spectrum of the recently described asparagine synthetase deficiency and show a remarkable clinical homogeneity among affected individuals, which should facilitate its recognition and molecular confirmation for pertinent and timely genetic counseling.

**Electronic supplementary material:**

The online version of this article (doi:10.1186/s12883-016-0633-0) contains supplementary material, which is available to authorized users.

## Background

Asparagine synthetase deficiency (ASNSD, OMIM# 615574) is a very rare newly described autosomal recessive neurometabolic disorder, caused by homozygous or compound heterozygous mutations in the *ASNS* gene on chromosome 7q21 [1]. The phenotype is characterized by microcephaly, severely delayed psychomotor development, progressive encephalopathy, cortical atrophy with reduced cerebral volume and enlarged lateral ventricles (associated in some with cerebellar and pontine hypoplasia, simplified gyral pattern, cortical dysgenesis and delayed myelination), intractable seizures or hyperekplexic activity, appendicular hypertonia and hyperreflexia [1]. Other associated features include micrognathia, receding forehead, relatively large ears, axial hypotonia and cortical blindness.

To the best of our knowledge, only 12 patients were described in the literature including the nine original patients reported by Ruzzo et al. [1] from four families of Iranian Jewish, French Canadian, and Bangladeshi origins, two of whom were consanguineous. Two patients were subsequently reported by Alfadhel et al. [2] from Saudi Arabia, and another reported by Ben-Salem et al. [3] from United Arab Emirates, all born to consanguineous parents (Table 1).

**Table 1:** Clinical features of cases with asparagine synthetase deficiency due to *ASNS gene* mutation

	Present report		Ruzzo et al. [1]	Ben Salem et al. [3]	Alfadhel et al. [2]
	Patient 1	Patient 2			
Number of pts	2		9	1	2
Number of families	2		4	1	1
Age	1 month	4 Yrs	9 month–14 Yrs	5 Yrs	2 Yrs/4 Yrs
Gender	M	M	8M/1F	M	1M/1F
Ethnic origin	Arab	Arab	Iranian Jews, French	Arab	Arab
			Canadian, Bangladeshi		
Consanguinity	Yes	Yes	Yes in two families	Yes	Yes
Mutation in *ASNS* gene	c.1219C > T	c.944A > G	c. 1084T > G(p.Phe 362Val	c. 1193 A > C p.Y 398 C	c. 1160A > G
	p.(Arg407)	p.Y 315 C	c. 1648C > T(p.Arg 550Cys		p. Tyr 377Cys
			c 17C > A(p. A6E		
Type of mutation	Nonsense homozygous	Missense, homozygous	Missense, homozygous, compound heterozygous	Missense, homozygous	Missense, homozygous
Developmental delay		Severe	Severe	Severe	Severe
Head circumference (cm )at birth	29	29	28.5–33	29.5	30 and 26.5
Hypertonia	No	Yes	Yes	Yes	Yes
Spastic quadriplegia	No	Yes	Yes	Yes	Yes
Seizure	Yes	No	6 patients	Yes	Yes, both
Hyperekplexia	Yes	Yes	Three patients	No	No
EEG Pattern	Epileptic encephalopathy in a transitional phase with predominant SZ burdens	Low amplitude bilaterally but no clear epileptiform discharge	• Disorganized background In hyperekplexia cases • Hypsarrhythmia • Suppression burst	Abnormal background activity bilaterally, low amplitude and frequent interictal multifocal spike	Multiple independent spike foci
MRI Brain	Microcephaly, smooth thin cerebral cortex, simplified gyral pattern, global brain atrophy, delayed myelination, hypoplastic cerebellum and pons	Microcephaly, smooth thin cerebral cortex, simplified gyral pattern, global brain atrophy, delayed myelination, hypoplastic cerebellum and pons	All have severe microcephaly, brain atrophy delayed myelination, decreased size of the pons and simplified gyral pattern	Severe microcephaly thin corpus callosum, ventriculomegaly, brain atrophy, decreased size of pons, simplified gyral pattern	Both severe microcephaly brain atrophy, delayed myelination and simplified gyral pattern

*Abbreviations*: *M* male, *F* female, *Yrs* years, *EEG* electroencephalography, *MRI* magnetic resonance image

In this report, we describe two additional cases from Saudi Arabia belonging to two consanguineous families, with typical clinical and radiological features of ASNSD. The diagnosis was confirmed by whole exome sequencing (WES), which revealed two novel mutations in the *ASNS* gene. This is the fourth ASNSD report in the literature.

## Case presentation

### Patient 1

**Fig. 1:**
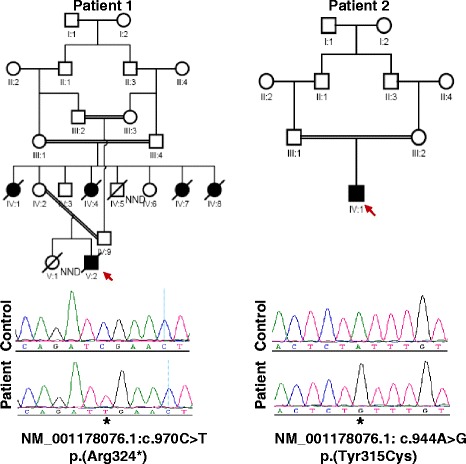
Pedigrees of the two study families. The sequence chromatograms of the mutant alleles are shown below the respective pedigrees

**Fig. 2:**
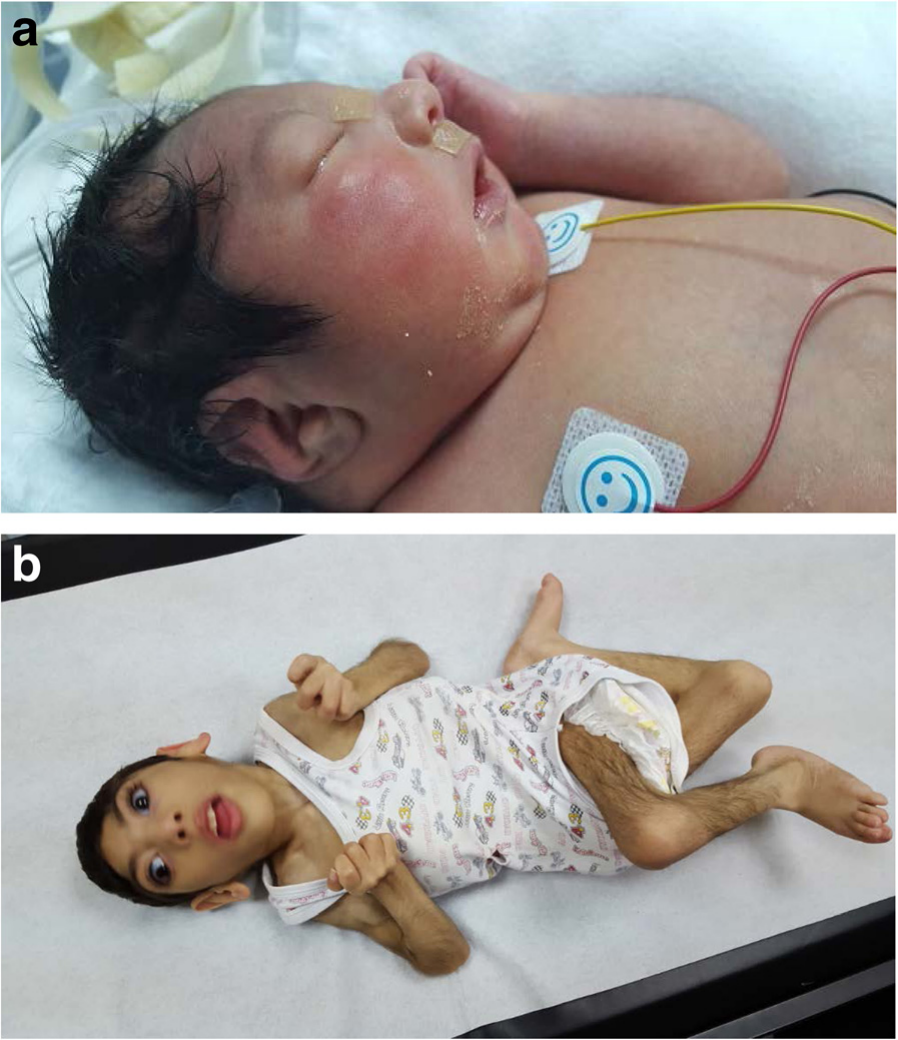
**a** Photograph of patient 1 showing microcephaly, slopingforehead, micrognathia, and relatively large ears. **b** Photograph of patient 2 at age of four years. Note microcephaly, relatively large ears, sloping forehead, and severe contractures of all limbs (spastic quadriplegic posture)

**Fig. 3:**
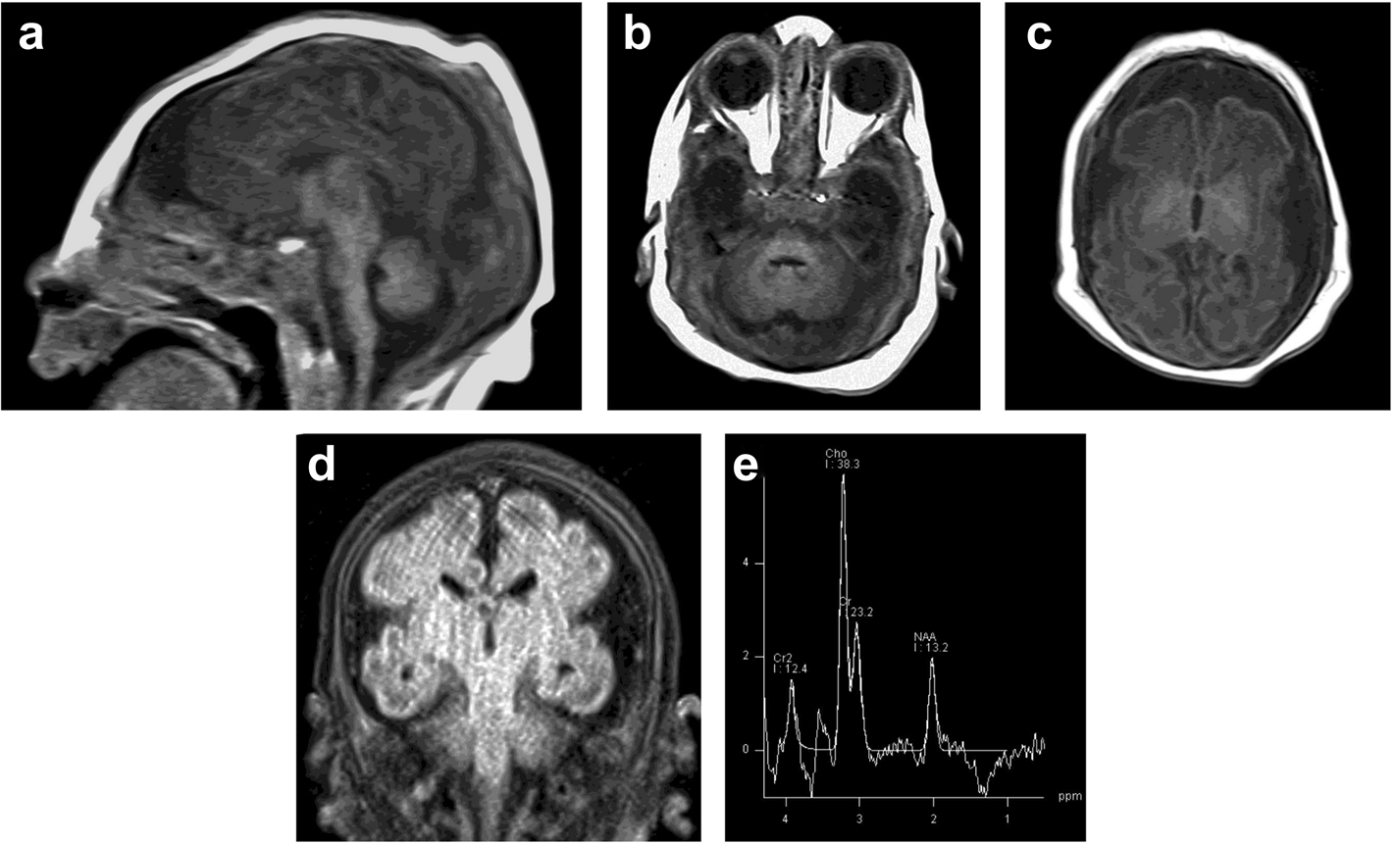
Brain MR images at day 1 after birth of patient 1. **a** Sagittal T1-weighted image showing severe microcephaly, simplified gyral pattern, thin corpus callosum, small cerebellum, and small pons. **b** Axial T1-weighted image demonstrating small pons and cerebellum. **c** Axial T1-weighted image showing delayed myelination of the posterior limb of internal capsule, bilaterally. **d** Coronal FLAIR image demonstrating severely simplified gyral pattern and large extra-axial CSF spaces reflecting brain underdevelopment. **e** Proton MR Spectroscopy with long TE showing normal spectra for age

The proband (Fig. 1, V: 2) is 1-month-old Saudi boy born normally at term to first 24-year- old parents. Four maternal aunts (Fig. 1, IV: 1, 4, 7, 8.) had died at the age of four, five, three and six weeks respectively, in a remote medical facility with no available records. However, all are said to have presented with microcephaly and abnormal movements similar to the index (see below). The mother was G2P1 (IUFD at 28 weeks gestation) +0. No history of exposures. Antenatal ultrasound (US) scan showed microcephaly. Apgar score was 9 and 10 at one and five minutes, respectively. Birth weight 2675gm (10^th^ percentile), head circumference 29 cm (−3SD). Examination showed microcephaly, sloping forehead, short neck, and micrognathia (Fig. 2a). Shortly after birth, he developed abnormal movements in the form of bursts of tonic/clonic movements provoked by non-habituating glabellar and root of the nose tapping, sound and light (see Additional files 1, 2 and 3). There was hyperreflexia, hypertonia and arthrogryposis of the lower limbs. He developed frequent apneas necessitating mechanical ventilation. Treatment with clonazepam was initiated and later phenobarbitone was added to control the abnormal movements. Laboratory investigations (Table 2) showed normal metabolic screen including plasma and CSF asparagine, glutamine, aspartate and glutamate.CSF neurotransmitters, 5HIAA, 3-OMD, and HVA were normal. Brain MRI (Fig. 3) revealed cerebral atrophy, simplified gyral pattern and hypoplastic cerebellum and pons. EEG showed multifocal discharges, fast spiking in the left hemisphere favoring cortical dysplasia, and frontal spikes. The findings favor epileptic encephalopathy in a transitional phase with predominant seizure burdens. Whole exome sequence (WES) revealed nonsense mutation in the *ASNS* gene, (NM_001178076.1: c.970C > T p. (Arg324*). He died at the age of six weeks in status epilepticus.

**Table 2:** Biochemical findings in patient 1

Test	Plasma	Reference range	Cerebrospinal fluid	Reference range
Asparagine (μ *mol*/L)	55	25–91	5	0–12
Glutamine (μ *mol*/L)	834	316–1020	639	232–725
Glutamic acid (μ *mol*/L)	125	31–202	2	0–27
Aspartic acid (μ *mol*/L)	33	2–20	<1	0–3
CSF Neurotransmitters				
5-hydoxyindoleacetic acid(5HIAA) (n mol/L)			281	Newborn (208–1159)
Homovanillic acid (HVA) (n mol/L)			410	Newborn (337–1299)
3-O-methyldopa (3-OMD) (n mol/L)			79	Newborn (0–300)
Urine organic acid	Normal

### Patient 2

Is a 4- year-old Saudi boy delivered normally at term to a 23-year-old primigravida lady and her 25-year-old first cousin husband (Fig. 1, IV:1). Antenatal US scan revealed microcephaly but pregnancy was uneventful otherwise. Apgar score was 9 and 10 at one and five minutes, respectively. Birth weight 2790 gm (25^th^ percentile), length 51 cm (50^th^ percentile) and head circumference 30 cm (−2.6SD). He was admitted to the Neonatal Intensive Care Unit (NICU) because of microcephaly and abnormal movements. Clinical examination showed microcephaly, staring anxious look, sloping forehead, receding chin and relatively large ears. Neurological examination revealed tonic/clonic rapid movements of both upper and lower limbs, with positive head retraction reflex (HRR). The attacks were provoked by glabellar and tip of the nose tapping, were non-habituating and precipitated by sounds. Additional files 4, 5, and 6 show this in more detail. Hypertonia with exaggerated reflexes and arthrogryposis of both upper and lower limbs were also noted. The rest of the systemic examination revealed no abnormality. He was initially managed by phenobarbitone. Clonazepam and levetiracetam were later added due to the intractable movements. At the age of four years he was found to have profound global developmental delay and spastic quadriplegia with severe contractures of both upper and lower limbs (Fig. 2b). He also had cortical blindness and hyperekplexic activities could still be elicited by glabellar and root of the nose tapping, light and sounds [see Additional files 1, 2, 3, 4, 5 and 6]. His growth parameters were severely retarded: his weight 5.3 Kg (−6.8SD) and head circumference was 35 cm (−10.1SD).

**Fig. 4:**
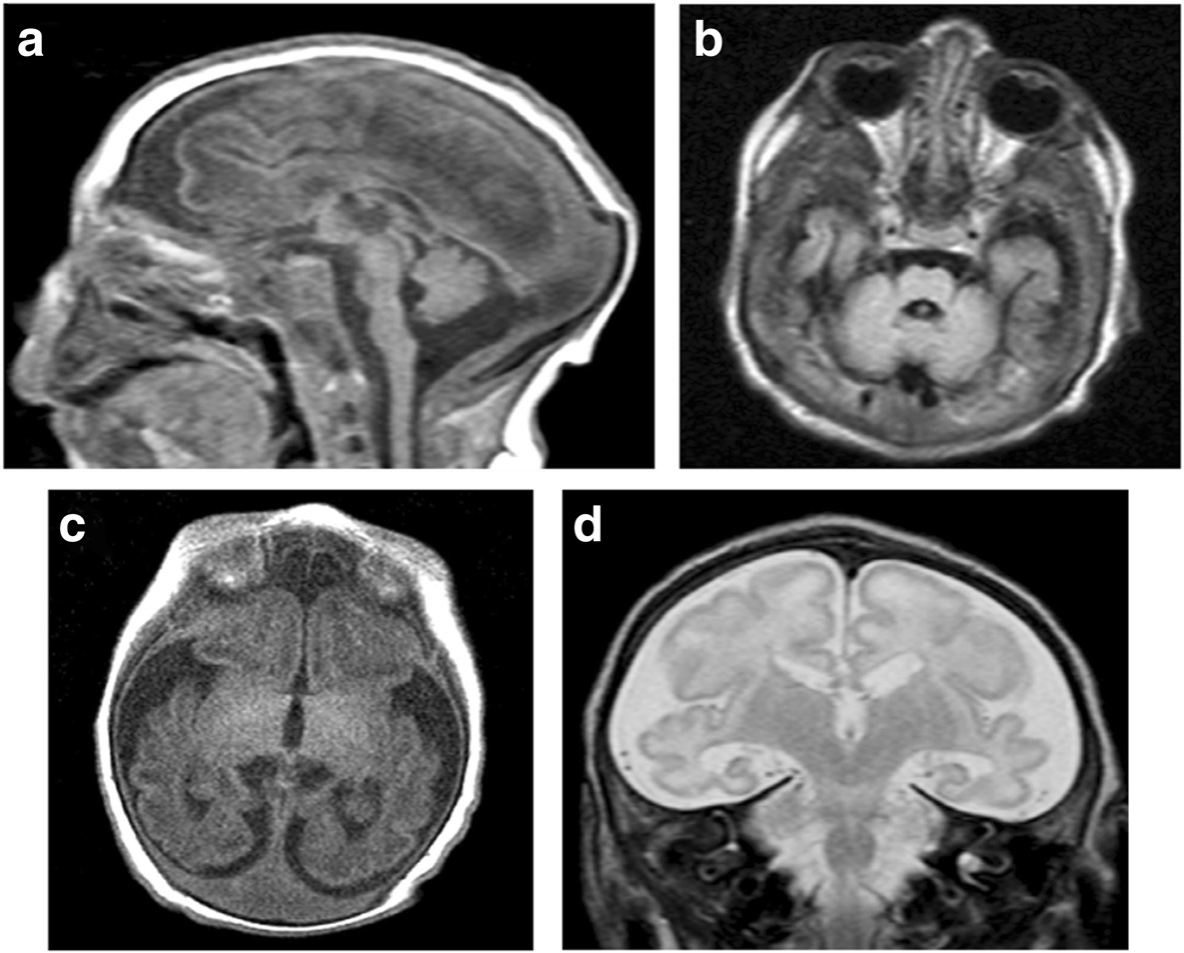
MRI brain at the age of 3 weeks. **a** Sagittal T1-weighted image showing severe microcephaly with overlapped lambdoid sutures, brain underdevelopment evident by simplified gyral pattern, small cerebellum, and small pons. **b** Axial FLAIR image revealing small pons and cerebellum. **c** Axial T1-weighted image demonstrating delayed myelination evident by absent myelination of the posterior limb of internal capsule, bilaterally. **d** Coronal T2-weighted image showing simplified gyral pattern (more in the frontal lobes) and large extra-axial CSF spaces due to brain underdevelopment

Laboratory tests including hematologic indices, renal function, liver function, and electrolytes were all normal. Metabolic screen, plasma amino acids, lactate, and ammonia were unremarkable. Chromosome study revealed normal male karyotype. MRI brain (Fig. 4) showed microcephaly, thin and smooth cortex with simplified gyral pattern [4], delayed myelination, dilatation of the ventricles, global brain atrophy and hypoplastic cerebellum and pons. EEG showed very low amplitude bilaterally without epileptiform discharges. Only sporadic sharp transient spikes, most likely myogenic in origin, were noted. Genetic testing, utilizing whole exome sequencing (WES), showed a novel homozygous *ASNS* gene mutation: NM_001178076.1, missense c.944A > G, *p. (*Tyr315Cys*)*. Both parents are heterozygous carriers. This change was absent in 615 in-house Saudi exomes, and is predicted pathogenic by PolyPhen (0.992), SIFT (1.0) and CADD (28.7) [5]. The family was offered genetic counselling.

We report two novel mutations in the *ASNS* gene in two Saudi patients from first cousin marriages who presented with congenital microcephaly, hyperekplexia, cerebral atrophy, simplified gyral pattern, and hypoplastic cerebellum and pons (Figs. 3 and 4). The phenotype is consistent with the recently described ASNSD (OMIM# 615574). *ASNS* encodes an asparagine synthetase enzyme involved in the synthesis of asparagine from glutamine and aspartate [6]. The neurological impairment resulting from *ASNS* mutation can be explained by asparagine depletion in the brain or by accumulation of aspartate /glutamate leading to enhanced excitation and neuronal damage [1]. To date five pathogenic mutations in the *ASNS* gene have been identified (Table 1).

All cases of asparagine synthetase deficiency were diagnosed by molecular genetics as there is no reliable biochemical test for diagnosing the disorder. Alfadhel et al. [2] reported low levels of asparagine in the CSF of two siblings with ASNSD confirmed by molecular genetics, while Ruzzo et al. [1] reported low asparagine levels in the CSF of only two of the five patients with the disorder. Our two patients had normal plasma and CSF asparagine, glutamine, aspartate, and glutamate and CSF neurotransmitters (Table 2). Therefore, this condition cannot be ruled out by normal plasma and CSF asparagine, aspartate and glutamate levels (Table 3) in a patient presenting with congenital microcephaly, and unexplained encephalopathy in the form of intractable seizures or hyperekplexia [1].

**Table 3:** Biochemical Findings in ASNSD

	Plasma				Cerebrospinal fluid (CSF)				
	Asparagine μmol/L	Glutamine μmol /L	Aspartate μmol /L	Glutamate μmol /L	Asparagine μmol /L	Glutamine μmol /L	Aspartate μmol /L	Glutamate μmol /L	Comment
A. Present Report (2015)									
Patient 1	55 (25–91)	834 (316–1020)	33 (2–20)	125 (31–202)	5 (0–12)	639 (232–725)	<1 (0–3)	2 (0–27)	Slightly Elevated Plasma Aspartate
Patient 2	Normal	Normal	Normal	Normal	N/A	N/A	N/A	N/A	
B. Majid Alfadhel et. al (2014) [2]									
Patient 1	10 (33–68.4)	339 (254–823)	N/A	N/A	Not detected (1.1–6.9)	922 (356–680)	N/A	N/A	Low Plasma and CSF Asparagine
Patient 2	6 (33–68.4)	328 (254–823)	N/A	N/A	1 (1.1–6.9)	574 (356–680)	N/A	N/A	Low Plasma and CSF Asparagine
C. Ruzzo el. al (2013) [1]									
1	57 (23–112)	1250 (254–823)	18 (1–24)	N/A	N/A	N/A	N/A	N/A	High Plasma Glutamate
2	49 (23–112)	1149 (254–823)	2 (1–24)	N/A	N/A	N/A	N/A	N/A	High Plasma Glutamate
3	N/A	N/A	7 (17–21)	N/A	N/A	N/A	N/A	N/A	Low Plasma Aspartate
4	12 (16–21)	N/A	N/A	N/A	N/A	N/A	N/A	N/A	Low Plasma Asparagine
5	N/A	N/A	12 (0–20)	N/A	N/A	N/A	N/A	N/A	
6	11 (31–56)	439 (474–736)	7 (4–18)	N/A	N/A	N/A	N/A	N/A	Low Plasma Asparagine
7	55 (31–56)	668 (474–736)	9 (4–18)	N/A	N/A	N/A	N/A	N/A	
Ben-Salem et. al (2015)	Normal	Normal	Normal	Normal	N/A	N/A	N/A	N/A	

Legend: N/A Not Available

ASNSD is a very rare disorder with a prevalence of <1/1000000 worldwide (ORPHA 391376); and hitherto, only 12 cases have been reported. We suspect this condition is underdiagnosed due to lack of recognition and the risk of confusing the hyperekplexia phenotype for nonspecific neonatal epilepsy, and also due to lack of a specific laboratory test and the difficulty of performing molecular genetic testing in suspected cases except in research centers or referral hospitals. Given that there is no reliable biochemical test, sequencing of ASNS is the mainstay of diagnosis. While our two patients were diagnosed using WES, this gene can easily be targeted by Sanger sequencing or added to a gene panel approach as described before [7]. The clinical and radiographic presentation of our patients, namely congenital microcephaly, hyperekplexia, brain malformation, and (in long term survivors) severe psychomotor retardation and cortical blindness is identical to that observed in the patients reported by Ruzzo et al. [1] with *ASNS* gene mutation. Interestingly, we previously [8] reported six patients with a similar presentation of hyperekplexia, microcephaly and brain malformations who later proved to have cathepsin deficiency (CTSD) known to be associated with congenital ceroid lipofuscinosis neuronal 10 (CLN 10) [9, 10]. Our observation raises the intriguing possibility of a link between asparagine synthetase deficiency and cathepsin deficiency, although this will require future research. Both disorders should be considered in microcephalic neonates who present with seizures or hyperekplexia at or before birth, and molecular genetic testing needs to be performed for pertinent and timely genetic counseling. This will pave the way for adopting effective preventive and therapeutic approaches like preimplantation genetic diagnosis (PGD), or early termination of affected pregnancies, which helped many families in this region with high prevalence of autosomal recessive disorders [11]. Therapeutic approach by supplementation with asparagine in ASNSD seems attractive. However, the prenatal onset of the microcephaly and early postnatal presentation make such treatment unlikely to be curative unless started prenatally [1].

## Conclusion

we expand the allelic heterogeneity of ASNSD and emphasize the clinical homogeneity of this disorder. The remarkable clinical overlap with CTSD-related CLN10 makes it difficult to segregate the two disorders clinically and highlights the need for ANSN and CTSD sequencing to make an accurate diagnosis.

## Abbreviations

3-OMD, 3-O-Methyldopa; 5HIAA, 5 hydroxy indole acetic acid; ASNS, asparagine synthetase; ASNSD, asparagine synthetase deficiency; CLN, ceroid lipofuscinosis neuronal; CSF, cerebrospinal fluid; CTSD, cathepsin D; EEG, electroencephalogram; HRR, head retraction reflex; HVA, homo vanillic acid; IUFD, intra uterine fetal death; MRI, magnetic resonance image; NICU, neonatal intensive care unit; PGD, preimplantation genetic diagnosis; SD, standard deviation; US, ultrasound; WES, whole exome sequence

## Additional files

Additional file 1: MOV showing non-habituating glabellar and root of the nose tapping in patient 1 (Fig. 1,V.2). (MP4 9415 kb) Additional file 2: MOV shows exaggerated startle reflex to sounds in patient 1 (Fig. 1,V.2). (MP4 8851 kb) Additional file 3: MOV shows exaggerated startle reflex to light in patient 1 (Fig. 1,V.2). (MP4 3117 kb) Additional file 4: MOV showing non-habituating glabellar and root of the nose tapping in patient 2 (Fig. 1IV .1), in the first week of life. (MP4 3050 kb) Additional file 5: MOV shows exaggerated startle reflex to sound in patient 2 (Fig. 1IV .1), at the age of 4 years. (MP4 3052 kb) Additional file 6: MOV shows exaggerated startle reflex to light in patient 2 (Fig. 1IV .1), at the age of 4 years. (MP4 2722 kb)
